# Functional Hydrogel Dressings for Treatment of Burn Wounds

**DOI:** 10.3389/fbioe.2021.788461

**Published:** 2021-12-06

**Authors:** Wentao Shu, Yinan Wang, Xi Zhang, Chaoyang Li, Hanxiang Le, Fei Chang

**Affiliations:** ^1^ Department of Biobank, Division of Clinical Research, The First Hospital of Jilin University, Changchun, China; ^2^ Key Laboratory of Organ Regeneration and Transplantation of the Ministry of Education, The First Hospital of Jilin University, Changchun, China; ^3^ Department of Burn Surgery, The First Hospital of Jilin University, Changchun, China; ^4^ Department of Orthopedics, The Second Hospital of Jilin University, Changchun, China

**Keywords:** burn wound, hydrogel dressing, emergency temporary coverage, antibacterial, stem cells, factors promoting wound healing

## Abstract

The therapy of burns is a challenging clinical issue. Burns are long-term injuries, and numerous patients suffer from chronic pain. Burn treatment includes management, infection control, wound debridement and escharotomy, dressing coverage, skin transplantation, and the use of skin substitutes. The future of advanced care of burn wounds lies in the development of “active dressings”. Hydrogel dressings have been employed universally to accelerate wound healing based on their unique properties to overcome the limitations of existing treatment methods. This review briefly introduces the advantages of hydrogel dressings and discusses the development of new hydrogel dressings for wound healing along with skin regeneration. Further, the treatment strategies for burns, ranging from external to clinical, are reviewed, and the functional classifications of hydrogel dressings along with their clinical value for burns are discussed.

## 1 Introduction

Between 2015 and 2019, 550,000 people worldwide died from fire, heat and hot substances, and 37 million people were disabled and requiring medical treatment (World Health Organization., 2019). Unfortunately, >95% of these burn injuries occur in low- and middle-income countries (Peck, 2011). Severe burns continue to pose a major challenge in regions with limited medical resources, especially developing countries. With the continuous improvement of burn care, development of new burn dressings is crucial.

## 2 Clinical Therapies for Burn Wounds

Thermal injury is one of the most severe and complex forms of trauma, and one of the main causes of disability. Thermal injury can be caused by heat, high-voltage electricity, or chemicals. People who suffer severe burns may suffer from severe emotional distress, which can lead to mental illness. Severe burns necessitate long-term hospitalization, which results in enormous nursing costs, and can be accompanied by a series of fatal complications (e.g., shock, electrolyte imbalance, respiratory failure, and wound infection). The three main risk factors of death for burn patients are old age, a non-superficial burn accounting for >40% of the total burn surface area (TBSA), and inhalation injury (American Burn Association Burn, 2016).

**FIGURE 1:**
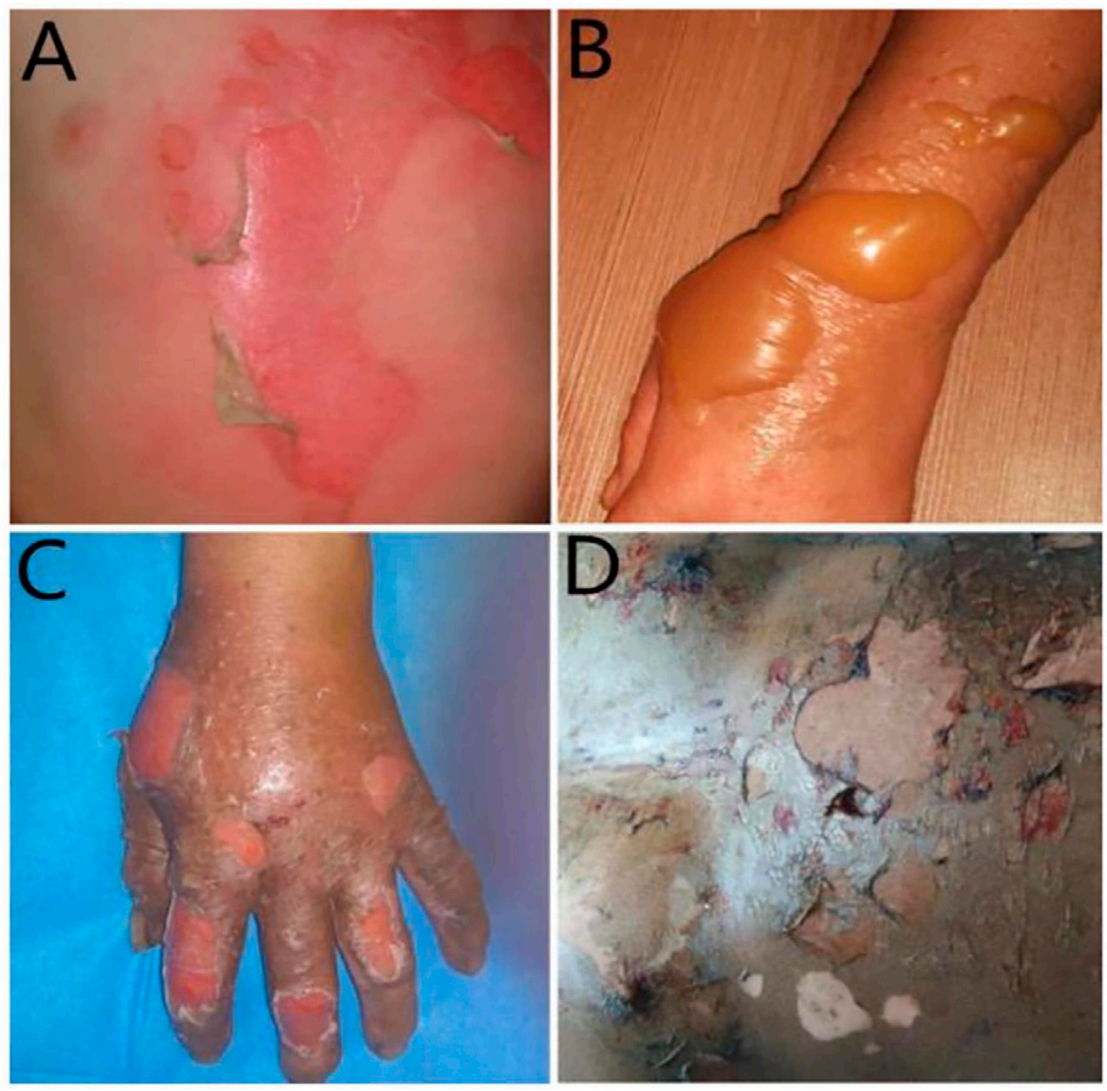
Burn wounds. **(A)** First degree. **(B)** Superficial second degree. **(C)** Deep second degree. **(D)** Third degree.

Evaluation of burn patients involves two crucial parameters: wound depth and TBSA (Burd and Yuen, 2005) (Figure 1). First-degree burns affect the superficial layer of the epidermis. Superficial second-degree burns affect the epidermis and dermis. Deep second-degree burns affect the entire epidermis and dermis. Third-degree burns affect the epidermis, dermis, and subcutaneous tissue (Mertens et al., 1997). Burns can also be evaluated based on TBSA by the nine-point method, and the Lund–Browder table can be used to measure the injured body surface area of the patient accurately (Hettiaratchy and Dziewulski, 2004). In general, large-area burns include mixed burns of different depths, and the depth of burns may change after the initial injury (Hettiaratchy and Papini, 2004). Clinically, it is difficult to judge the wounds of deep second- and third-degree burns. Laser Doppler imaging is expected to be a powerful tool for evaluating burn depth (Jaskille et al., 2009; Jaskille et al., 2010).

The systemic treatment of burn patients is divided into preliminary assessment, emergency treatment, fluid resuscitation, severe management, and infection prevention.

Initial assessment comprises: identification of respiratory distress and smoke-inhalation injury; assessment of cardiovascular status and signs of shock; identification of injury complications; determination of burn severity. Emergency treatment of burns involves stabilizing the airway and supporting breathing. An essential component of systemic therapy is fluid resuscitation, which should be started <2 h after the burn has been sustained (Barrow et al., 2000). Management of severe burns involves meeting the high metabolic and nutritional needs of burn patients, provision of analgesia, sedation, and relieving anxiety; the early use of enteral nutrition and proton-pump inhibitors is important to prevent acute gastric ulcers (Raff et al., 1997). Sepsis prevention is critical in treating patients with severe burns, and systemic antibacterial drugs must be used cautiously to prevent the emergence of drug-resistant bacteria (Salick et al., 2007). The death of patients with large burn areas (>30% TBSA) is related to fungal infection of wounds, which requires additional fungal examination and use of antifungal drugs (Horvath et al., 2007).

### 2.1 Surgical Treatments

#### 2.1.1 Debridement and Escharotomy

Debridement and escharotomy are the main means to control wound infection. The debridement and excision of contaminated necrotic tissue and eschar improve the visibility of the wound surface (Latenser, 2009; Sen et al., 2009), prevent fragile eschar from being stretched and causing pain in damaged skin areas, and improve the limb ischemia caused by large-area eschar contraction (Dries, 2005). Most importantly, necrotic tissue and eschar are “hotbeds” for the growth and reproduction of bacteria. Removing them minimizes the hidden danger of wound infection and facilitates direct treatment of the wound with therapeutic drugs (Bishop, 2004; DeSanti, 2005).

#### 2.1.2 Skin Transplantation

Skin transplantation is a common method for closing burn wounds. For stable patients with small burn areas and a sufficient source of skin, autologous skin grafts of medium thickness are often used (Hermans, 2014). For burn patients with large burn areas and/or insufficient donor skin, temporary covering with allografts, xenografts, or skin substitutes is needed. Allografts are the optimum substance for the temporary sealing of large, life-threatening burns in cases with insufficient donor skin (American Association of Tissue Bank, 2016). However, cadaveric skin grafts have disadvantages: a serious risk of transmission of infectious disease, rejection, as well as difficulties in obtaining and storing cadavers. Xenotransplantation has a larger application space than that using autotransplantation, with more donors and larger available sizes. Xenografts are available from frogs, rabbits, dogs, and pigs. Nevertheless, natural xenografts often result in hypersensitivity, can spread zoonotic diseases, and are often rejected by the host. The development of specially treated xenografts needs major research.

### 2.2 Non-surgical Treatments

#### 2.2.1 Wound Dressings

**FIGURE 2:**
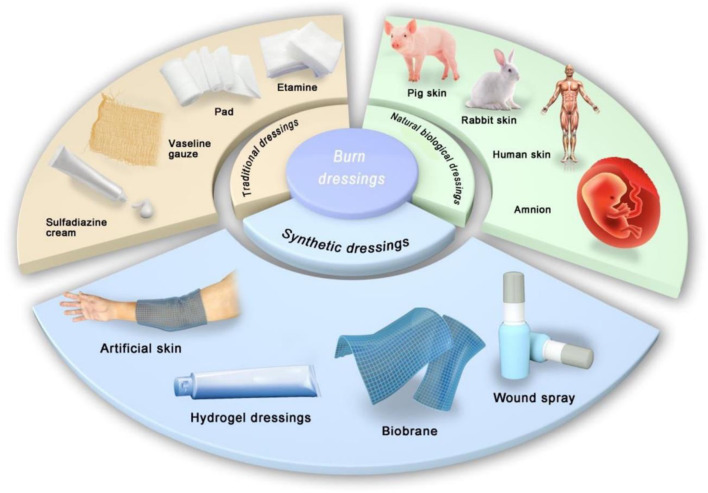
Classification of burn dressings: Traditional dressings, Natural biological dressings, and Synthetic dressings.

Dressings are applied to cover burn wounds, promote epithelialization, prevent infection and mechanical trauma, keep wounds moist, and reduce pain. Various types of dressings are available (Figure 2). The dressing materials can be made into films, foams, composites, sprays, and gels depending on the requirement. Traditional dressings such as Vaseline™ gauze, silicone tablets, and paraffin dressings (e.g., Mepitel^®^) are used commonly for daily care of burns. Silver-containing dressings such as Acticoat^®^, Mepilex Ag^®^, and Aquacel^®^ Ag have stronger antibacterial activity than silver sulfadiazine (SSD) cream, along with fewer adverse reactions and lower cost. Thus, these silver-containing dressings aid in the fight against wound infections (Tredget et al., 1998; Cuttle et al., 2007; Abboud et al., 2014). Biological dressings such as human amnion and processed products of dehydrated amnion chorionic villi overcome the shortcomings of acquisition of fresh amnion: inconvenience of processing and storage, high cost, and transmission of infectious diseases (Wasiak et al., 2013). Various synthetic dressings, alginate dressings, hydrocolloid dressings, hydrogel dressings, and polyurethane films (e.g., Mepilex^®^, DuoDERM^®^, Omniderm^®^) are used widely to cover the wound surface during re-epithelialization.

#### 2.2.2 Skin Substitutes

Biosynthetic skin and bioengineered skin substitutes, both self-cultivated and non-self-cultivated, are used for physiological wound closure until the epidermal layer is repaired. Biosynthetic skin, such as Biobrane^®^ (Smith & Nephew, London, United Kingdom), is a semi-synthetic double-layered material comprising a dermal analog (porcine collagen) and an epidermal analog (silicone resin), which is used to seal superficial burns temporarily. The wound surface and skin donor area are covered temporarily. Biological skin substitutes include GraftJacket^®^ (KCI, San Antonio, TX, United States), Alloderm^®^ (LifeCell, Bridgewater, NJ, United States), and Integra^®^ (Integra LifeSciences, Plainsboro, NJ, United States). Integra^®^ is a biosynthetic skin scaffold composed of a dermal layer (bovine collagen) and an epidermal layer (silicone membrane). Integra^®^ can reduce the size of the donor area, improve skin elasticity, and improve the appearance of burn wounds (Danin et al., 2012). However, skin substitutes are fragile, expensive, and exhibit poor anti-infection ability. Only experienced and highly trained surgeons can apply these products to patients with severe burns (Purdue et al., 1997). In recent years, the development of a new type of high-efficiency “compound skin” has been a research focus; scholars are eager to find a skin substitute with a similar appearance and texture to normal skin, good tissue elasticity, and friction resistance.

## 3 Advantages of Hydrogel Dressings

Wound healing involves numerous physiological processes. Burn dressings must have biocompatibility, biodegradability, a porous structure, and appropriate mechanical properties (Quinn et al., 1985). The characteristics of an ideal burn dressing are: 1) good adhesion to the wound surface (while not adhering to the wound surface) and remaining accessible for removal (Thomas et al., 1995); 2) transparent, visible, and non-enclosed with a reasonable rate of water-vapor transmission to prevent wound immersion; 3) provide a good barrier against bacterial invasion to prevent infection (Reithofer et al., 2014); 4) good biocompatibility, wide availability, and low cost. Although no material is perfect, hydrogel dressings have shown considerable advantages over traditional dressings (Selig et al., 2012).

Wound healing is a complex physiological process broadly divided into four phases: hemostasis, inflammation, proliferation, and degeneration. Compared with other wounds, the burn wound healing process is more complicated and affected by many factors. A large amount of devitalized tissue in the burn wound bed is caused by high temperature, which leads to a massive proliferation of pathogenic bacteria at the wound site and finally causes local or systemic infection. Considering the characteristics of burn wounds, the primary strategies for their treatment focus on restoring skin barrier function, reducing infection, and inhibiting scar formation and skin contracture. In clinical treatment, the autologous skin graft is generally considered the gold standard for burn treatment. But in cases of extensive severe burns, the source of autografts may be limited due to the unavailability of donor areas, skin contractures. Therefore, wound dressings have broad application prospects in the treatment of burn wounds. An ideal wound dressing should have suitable tack, absorbable rows, antimicrobial capacity, ease of replacement, and individually tailored size. In the process of burn treatment, hydrogel dressings have higher advantages. The modification of hydrogels can enable them to possess a variety of biological functions to fit the different needs that make the wound healing. The hydrogel dressing can control wound infection and perform autolytic debridement of necrotic tissue by piggybacking on antibiotics or anti-inflammatory drugs during the inflammatory phase. In the proliferative phase, hydrogel dressings can promote vascular regeneration and fibroblast proliferation by releasing growth factors and degradation of bioactive materials. In addition, hydrogel dressings can also be used as tissue engineering scaffolds to piggyback on seeded cells or induce factors to promote tissue generation. The porous structure possessed by the hydrogel can absorb wound exudates, maintain the excellent permeability and moist wound environment around the wound, and reduce the pain of patients to a certain extent. Therefore, the hydrogel dressing may serve as a new strategy for burn wound management and play an essential role in wound healing.

Hydrogel is a kind of water-rich polymer network, which is composed of natural or synthetic polymers. Hydrogel polymers are combined by various cross-linking methods to produce different functions and properties. The cross-linking methods are mainly divided into physical cross-linking and chemical cross-linking. Physical cross-linking mainly includes intermolecular interactions, such as hydrogen bonding, ion interaction, crystallization cross-linking, hydrophobic association and so on. The main characteristics of physically crosslinked hydrogels are low molecular toxicity and high biocompatibility. Chemical crosslinking is usually connected by covalent bonds between polymers, such as free radical polymerization crosslinking, radiation crosslinking and so on. Therefore, chemically crosslinked hydrogels have better mechanical properties.

Hydrogels have hydrophilic and soft tissue-like properties. They exhibit mixing behavior with mechanical properties similar to solids but diffusion characteristics resembling those of liquids. Thus, hydrogels can absorb and release water in a reversible manner in response to specific environmental stimuli (e.g., temperature, pH, ionic strength). This intelligent response to physiological variables determines how hydrogel dressings can be applied in the treatment of burn wounds.

Application of hydrogel dressings to treat burn wounds has three advantages. First, hydrogel dressings can absorb wound exudate; the amount of water absorbed by a hydrogel is thousands of times its dry weight (Hoffman, 2002). Moreover, the liquid-supply characteristics of hydrogels also help maintain a moist environment during wound healing, which is particularly important when treating dry wounds. Second, hydrogel dressings can be customized into any shape according to the wound condition. Third, hydrogel dressings can adhere to wounds without adhesion, as well as reduce the temperature and pain of the wound. They are also transparent, allowing the wound to be observed. Research on hydrogel dressings for burns is booming worldwide, and many achievements have been made (Figure 2). Hydrogel dressings account for most of the global market for dressings (USD 3 billion) (Kamoun et al., 2017).

## 4 Hydrogel Dressings for Burn Wounds

Multiple influencing factors need to be considered during the clinical treatment of burn injuries. First, the breakdown of the skin barrier and coverage of necrotic tissue after burn injuries make the burned tissue vulnerable to infection. Second, hyperthermic injuries often cause increased capillary permeability at the wound site, resulting in massive tissue fluid leakage from the damaged area. Depending on the deep part of the burn, the damage can spread to the dermal tissue, and resulting in several complications.

Burn wound healing is a complex dynamic physiological process that involves several factors, including the regulation of inflammatory factors, cell migration and proliferation, and extracellular matrix deposition. Hydrogels with high hydrophilicity, good biocompatibility, and suitable pore structure can meet the demand in the burn wound healing process. Especially in treating irregular wounds, hydrogel dressings can form a good coverage and filling of the wounds. According to the clinical demand for burn wounds in different periods, hydrogel dressings are designed and play therapeutic roles mainly from three aspects: preventing infections, promoting repair, and constructing scaffolds for skin tissue engineering. In preventing infections, hydrogel dressings can provide better coverage of the wound surface and insulate the wound from the external environment. Second, the functionalized modification on the hydrogel can possess a specific antibacterial function to inhibit bacterial proliferation at the local site of damage. In the process of wound tissue regeneration, hydrogels can be used as a carrier of growth factors to achieve sustained release of growth factors in the wound bed, which is more conducive to tissue repair. In the study of constructing engineered skin tissue *in vitro*, the hydrogel can also serve as a template for tissue regeneration, providing a method to repair full-thickness burn wounds.

**FIGURE 3:**
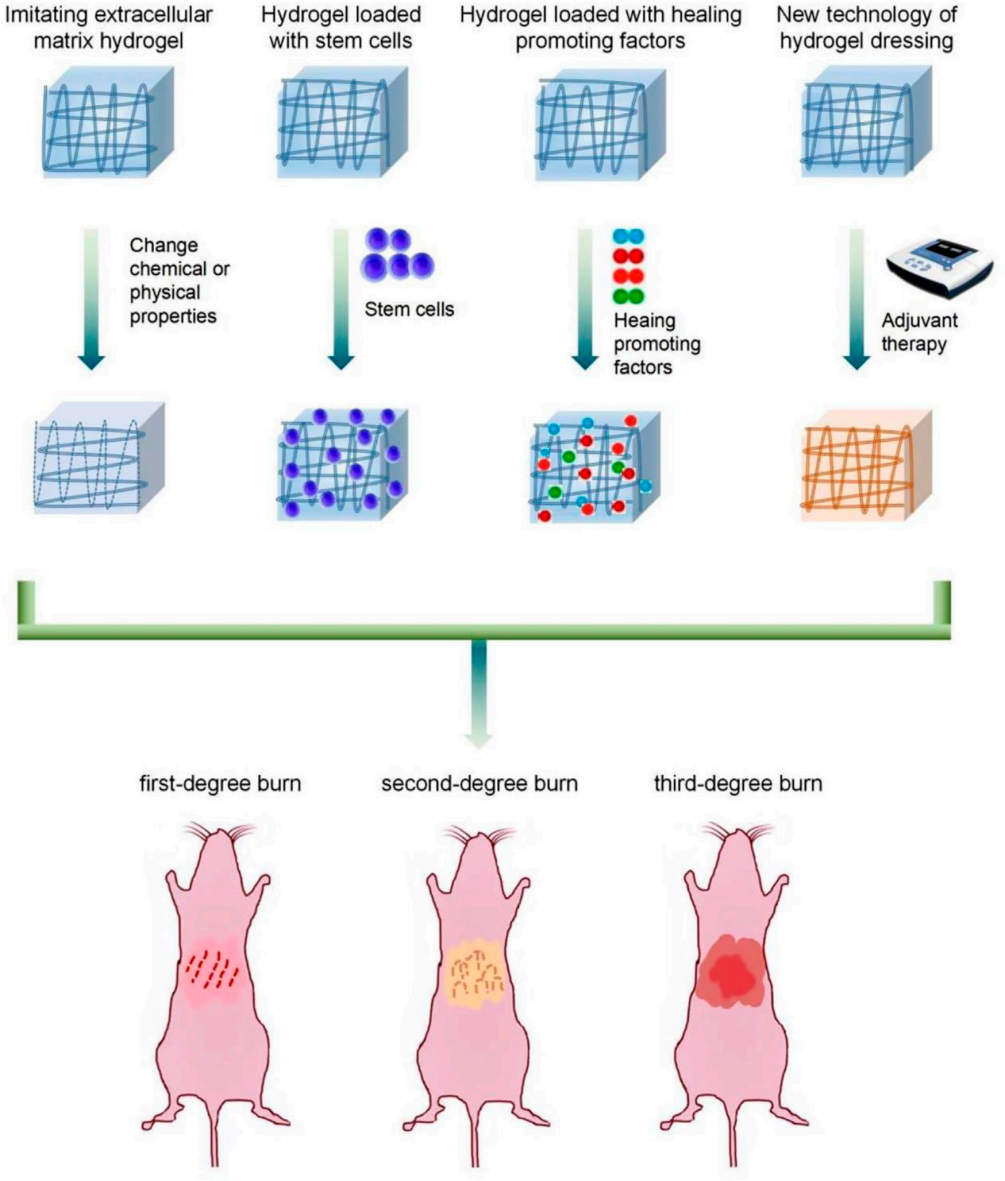
Functional classification of hydrogel dressings for burns: Imitating extracellular matrix hydrogel, Hydrogel loaded with stem cells, Hydrogel loaded with healing promoting factors, and New technology of hydrogel dressing.

The biocompatibility, biodegradability, and bioactivity of natural biopolymers (e.g., collagen, chitosan, cellulose acetate, gelatin, fibrin, hyaluronic acid (HA) and its salts, alginate) determine their potential value as dressings for burns. Sodium alginate has been shown to promote the proliferation of mouse fibroblasts. Collagen and polyose materials can also be used for the healing of burns. Collagen protein is one of the main components of extracellular matrix (ECM). It has been used widely in wound healing and is beneficial for treating severe burn wounds, pressure sores, and diabetic foot ulcers (Mozalewska et al., 2017). Hydrogel dressings made from several natural polymers have also been commercialized (Teodorescu et al., 2010). Compared with natural polymers, hydrogels from synthetic polymers have excellent controllable mechanical properties and biodegradability, along with lower cost and more abundant sources of raw materials. They can also be produced by various manufacturing technologies to provide a wide range of properties (Ekenseair et al., 2012). The combination of natural and synthetic materials can reduce their respective limitations and improve the efficacy of the resulting hydrogel dressing. Several types of hydrogel dressings are available, some of which contain additional drugs with anesthetic, anti-inflammatory, or nutritional properties (Figure 3).

Numerous hydrogel-based products are available for burn care, but research and development of hydrogel dressings continue unabated. *In situ*-molded gels and radiation-crosslinked gels have been introduced as new types of burn dressings (Sinko et al., 2015; Mohd Zohdi et al., 2012). In the future, hydrogel dressings for burns will consider the characteristics of wounds in various stages as part of a systematic, multifunctional strategy for the diagnosis and treatment of burns (Yan et al., 2012; Dargaville et al., 2013; Boonkaew et al., 2014) (Table 1).

**TABLE 1:** Summary of commonly employed polymers in hydrogel dressings and their functions in wound management.

Function	Main polymer	Bioactive agent	Burn depth	Function in wounds	Ref.
First aid	Carbomer 940		Full-thickness burn wounds	Nontoxic, improves tissue perfusion, reduces area of necrotic tissue in burn wounds	Chouhan, Lohe, Samudrala and Mandal, (2018)
	Natural silk fibroin		Full-thickness burn wounds	Promotes wound healing, facilitates the transition from the inflammation stage to the proliferation stage	Dang, Nguyen, Tran, Doan and Tran, (2018)
	Hydrazidemodified hyaluronic acid (HAAD)/benzaldehyde-terminated F127 triblock copolymer		Deep partial-thickness burn models	Adaptable mechanical strength, self-healing, liquid absorption/drainage, tissue adhesion, promotes repair of burn wounds	Li, Zhou, Li, Lin, Chen, Liu and Chen, (2018)
	Bacterial cellulose/acrylic acid (BC/AA)		Skin-burn wounds	Non-toxic, promotes wound-healing, enhances epithelialization, accelerates fibroblast proliferation	(Uppuluri and Shanmugarajan, 2019; Lei et al., 2020)
	Amphiphilic chitosan-g-pluronic copolymer	Curcumin	Second- and third-degree burn-wound models	Enhances regenerated collagen density, results in the formation of a thicker epidermis layer, increases collagen content, improves granulation, increases wound maturity, and enhances wound closure	Liang, Chen, Wang, Liu, Wei and Zhang, (2019)
	Dextran		Full-thickness burn wounds	Promotes neovascularization and skin regeneration	(Shen, Song, Papa, Burke, Volk and Gerecht, 2015; Stubbe, Mignon, Declercq, Van Vlierberghe and Dubruel, 2019)
	MPEG-PCL micelles/α- cyclodextrin	Dexamethasone sodium phosphate/Avastin^®^	Alkali-burn models in rats	High ocular biocompatibility and non-irritating after topical instillation, attenuates alkali burn-induced corneal inflammation, suppresses corneal neovascularization	Zhai, Xu, Zhou and Jing, (2018)
	Polyvinyl alcohol/agar	Icariin	Full-thickness burn wounds	Promotes new translucent skin tissue, repairs the ECM, enhances wound healing	Mohamad et al. (2014)
	HA/CCS/HLC		Skin burns on the backs of rabbits	Prevents bacterial infection effectively, promotes burn-wound healing better than a commercial film (DuoDERM^®^)	Pandey et al. (2017)
	CMC-Na/SA/CS		Second-degree burn wounds	Excellent self-regulatory and anti-adhesive properties that promote the healing of burn wounds synergistically	Dang, Nguyen, Tran, Doan and Tran, (2018)
	Gelatin/alginate		Burn wounds on skin	Good biocompatibility and cell-attachment properties	Sun, Zhang, Shen, Sebastian, Dickinson, Fox-Talbot, Reinblatt, Steenbergen, Harmon and Gerecht, (2011)
	Lysine-based dendron/PEG		Wounds from second-degree burns	Dissolves “on demand” through the thiol–thioester exchange reaction, allowing the burn dressing to be removed readily	Konieczynska, Villa-Camacho, Ghobril, Perez-Viloria, Tevis, Blessing, Nazarian, Rodriguez and Grinstaff, (2016)
	CMC/rigid rod-like dialdehyde-modified cellulose nanocrystal		Skin-burn wounds	Injected into an irregular deep burn wound, it reforms rapidly into an integrated piece, completely filling the wound area. Amino-acid solution can be used to dissolve the hydrogel, allowing painless dressing removal	Huang, Wang, Huang, Wang, Chen, Zhang and Zhang, (2018)
	Keratin-chitosan	Nano-ZnO	Skin-burn wounds	Increases swelling, exhibits bactericidal activity	Huang, Wang, Yu, Yu, Zheng, Peng, He, Zhao, Zhang, Li and Wang, (2017)
Anti-bacterial	Silver sulfadiazine-bFGF		Partial-thickness burn wounds(Zhu, Jiang, Liu, Yan, Feng, Lan, Shan, Xue and Guo, 2018)	Non-toxic and safe	Chakrabarti, Islam, Hazarika, Mazumder, Raju and Chattopadhyay, (2018)
	MC	Silver oxide NPs	Second-degree burns	Excellent antimicrobial activity and healing of burn wounds	Dang, Nguyen, Tran, Doan and Tran, (2018)
	Agarose	Minocycline/gentamicin	Porcine burn models	Reduces burn depth and the number of bacteria	Grolman, Singh, Mooney, Eriksson and Nuutila, (2019)
	Poloxamer (F68/F127)	Boron	Second-degree burn wounds	Increases wound closure *via* fibroblast activity, induces vascularization, exhibits antimicrobial effects against bacteria	Demirci, Dogan, Karakus, Halici, Topcu, Demirci and Sahin, (2015)
	Chitosan	Moxiflfloxacin	Animals with bacterial loads	Shows better efficacy than conventional gels in *S. aureus*-infected burn wounds	Chhibber et al. (2020)
	Dextran-hyaluronic acid	Sanguinarine	Full-thickness burn infection model (MRSA, *E. coli*)	Improves re-epithelialization, enhances ECM remodeling	Zhu, Jiang, Liu, Yan, Feng, Lan, Shan, Xue and Guo, (2018)
	Hyaluronic acid/chondroitin sulfate/asiatic acid	Zinc oxide/copper oxide	Second-degree burn wounds	Non-toxic; exhibits significant antibacterial activity, promotes re-epithelization, collagen-fiber arrangement, and angiogenesis; shows significant wound-healing activity	Thanusha et al. (2018)
	Glycol chitosan	Colistin	Burn-infection model in animals *in vivo*	Performs almost as well as native colistin against colistin-sensitive and colistin-resistant *P. aeruginosa* strains	Zhu, Zhao, Kempe, Wilson, Wang, Velkov, Li, Davis, Whittaker and Haddleton, (2017)
	Carbomer	Ciprofloxacin and lidocaine	Models of second-degree burns	Reduces the wound-healing period, increases the number of fibroblasts, increases the rates of epithelialization and dermis reconstruction, has an immediate anesthetic effect	Sanchez, Breda, Soria, Tartara, Manzo and Olivera, (2018)
	Collagen	*Saccharomyces cerevisiae*	Full-thickness burn wounds	Improves the morphological and biomechanical characteristics of burn wounds	Oryan, Jalili, Kamali and Nikahval, (2018)
	Chiosan	Marine peptides	Burn wounds on the backs of rabbits	Enhances cell migration and promotes skin regeneration	Ouyang, Hu, Lin, Quan, Deng, Li, Li and Chen, (2018)
Loading with stem cells	BC/AA	Human epidermal keratinocytes/human dermal fibroblasts	Burn wounds in thymic-free mice	Has significant collagen deposition	Badiavas, (2004)
	Polysaccharide	MSCs	Alkali burns to the corneas of rats	Enhances the migration rate of primarily cultured corneal epithelial cells; improves the recovery of the corneal epithelium; reduces inflammation, neovascularization, and opacity of the healed cornea	Ke, Wu, Cui, Liu, Yu, Yang and Li, (2015)
	UArg-PEA (chitosan derivative)	MSCs	Wounds from third-degree burns	Promotes wound closure, re-epithelialization, granulation-tissue formation, and vascularization	Alapure, Lu, He, Chu, Peng, Muhale, Brewerton, Bunnell and Hong, (2018)
	Chitosan/collagen/β-glycerophosphate	MSCs	Wounds from third-degree burns	Shortens healing time, limits the inflammation area, enhances re-epithelialization, promotes formation of high-quality, well-vascularized granulation tissue, attenuates formation of fibrotic, and hypertrophic scar tissue	Zhou, Li, Zhang, Shi, Li and Ju, (2019)
	PEGylated fibrin chitosan/microspheres (SSD-CSM-FPEG)	Silver sulfadiazine	Burn wounds on rats infected with *P. aeruginosa*	Reduces bacterial infection and promotes neo-vascularization with improved matrix remodeling	Banerjee, Seetharaman, Wrice, Christy and Natesan, (2019)
Loading with wound healing-promoting factors	Hyaluronic acid, dextran, and β-cyclodextrin	Resveratrol/VEGF plasmid	Model of splinted excisional wounds in rats	Inhibits the inflammatory response and promotes microvascular formation while being biocompatible	Wang, Huang, Hu, Yang, Lan, Zhu, Hancharou, Guo and Tang, (2019)
		Recombinant human granulocyte/macrophage colony-stimulating factor	Deep partial-thickness burn wounds	Promotes healing effectively	Yan et al. (2012)
Others	Rabbit collagen	Human amnion	Second-degree burns in rats	Non-cytotoxic, accelerates wound healing based on complete re-epithelialization and closure by wound contraction	Rana, Rahman, Ullah, Siddika, Hossain, Akhter, Hasan and Asaduzzaman, (2020)
	Honey		Burn-induced wounds in mice	75% honey–chitosan hydrogel possesses greater wound healing activity compared with that of commercial treatment and can be used safely as an effective natural treatment for topical wound healing	Mohd Zohdi et al. (2012)
	Sea cucumber		Burn-induced wounds in mice	Stimulates tissue regeneration and regulation of pro-inflammatory cytokines	Zohdi et al. (2011)
	Alginate	PWD	Burn-induced wounds in pigs	Safely delivers high concentrations of antibiotics in a hydrogel and treats burn infections	Nuutila, Grolman, Yang, Broomhead, Lipsitz, Onderdonk, Mooney and Eriksson, (2020)

### 4.1 First Aid Hydrogel Dressings

Several studies have reported new wound dressings for pre-hospital emergency treatment, and there is a large market demand for these dressings. In recent years, there has been a rapid increase in the use of alternative emergency cooling and dressings for burn patients in pre-hospital settings. In the United Kingdom, 39% of emergency medical services use burn dressings as first-aid coolants (Walker et al., 2005). Nearly 80% of British fire departments use hydrogels as cooling dressings (Cuttle et al., 2009). One study in Australia found that 13% of pediatric patients received first aid involving burn dressings (Hyland and Harvey., 2014). In a cohort study of 455 people, Hyland and colleagues (Hayati et al., 2018) found that >50% of patients were treated with hydrogel products by non-professional first-aid personnel. In the pre-hospital environment, the lack of skin coverage is the greatest threat to severely burned patients. To reduce fluid loss and prevent bacterial infection, hydrogel dressings are needed urgently to cover burn wounds.

Carbomer 940 hydrogel is a simple, low-cost dressing for burns that can improve tissue perfusion and decrease the area of necrotic tissue in burn wounds (Chouhan et al., 2018). *In situ*-formed hydrogels generated from natural silk fibroin separated from *Bombyx mori* along with *Antheraea assama* support the proliferation of primary human dermal fibroblasts and keratinocyte migration. They also provide support for full-thickness, third-degree burn wounds (Dang et al., 2018). The novel hydrogel HA-az-F127 is formed by the reaction between hydrazide-modified HA and benzaldehyde-terminated F127 triblock copolymer. HA-az-F127 exhibits rapid gelation and shear-thinning behavior (Li et al., 2018). It also exhibits high adaptability with regard to mechanical strength, good self-healing, and the ability to promote wound-tissue repair on a deep partial-thickness-burn model. Bacterial cellulose hydrogels are becoming increasing popular due to their biocompatibility. Bacterial cellulose/acrylic acid (BC/AA) hydrogels can improve the healing rate of burn wounds, accelerate fibroblast proliferation significantly, and promote wound epithelialization (Uppuluri and Shanmugarajan, 2019; Lei et al., 2020). Oxygen-free radicals are considered important factors for inhibiting wound healing, whereas curcumin is an effective antioxidant and anti-inflammatory agent. Liang et al. (Liang et al., 2019) used curcumin and amphiphilic chitosan-g-pluronic copolymer to create an injectable nanocomposite hydrogel (nCur-CP hydrogel). When applied to burn wounds, nCur-CP hydrogel showed higher collagen content, better granulation, and higher wound maturity in models of second- and third-degree burn wounds.

Neovascularization is an important factor affecting wound healing after severe burns. Sun and coworkers (Sun et al., 2011; Stubbe et al., 2019) found that dextran (Dex)-based hydrogels can be used in third-degree burn wounds, and promote the regeneration of blood vessels and skin in the wound. Conversely, in a model of alkali burns to the cornea, loss of vision along with graft rejection are closely related to corneal neovascularization. Huang and collaborators (Huang et al., 2018) prepared a supramolecular hydrogel comprising MPEG-PCL micelles and α-cyclodextrin that can co-deliver dexamethasone and Avastin^®^ in a burn model in rats. Corneal inflammation significantly inhibits the formation of corneal neovascularization. The supramolecular hydrogel was also found to have a significant prolongation effect on Avastin^®^.

To deal with the destruction of local-skin function after burns and the loss of a large amount of water and electrolytes from the wound surface, hydrogel dressings have been explored to repair the ECM. Some scholars believe that icariin can repair the ECM after burn injury. When an icariin-loaded polyvinyl alcohol/agar hydrogel was used for burn treatment, new translucent skin tissue appeared on the wound surface and the ECM was repaired (Mohamad et al., 2014). Pandey et al. (2017) mixed HA, carboxylated chitosan, and human-like collagen to simulate the ECM and used glutamine transaminase as a crosslinking agent to optimize the mechanical properties and pore size of the hydrogel. The resulting hydrogel was more conducive to burn wounds compared with the film (DuoDERM^®^). Few dressings used for burns can regulate humidity to optimize recovery. Liang et al. (2018) constructed a water-soluble carboxymethylcellulose sodium/sodium alginate/chitosan composite hydrogel with excellent self-regulatory ability and anti-adhesive properties that synergistically promoted the healing of burn wounds in rats. Sun et al. (2011) combined photocrosslinkable functionalities with hydrogel films to modify gelatin and alginate. The resulting products showed better cell adhesion and superior mechanical properties. Hydrogels that can be dissolved “on demand” are also needed in clinical settings. A dissolvable gel dressing does not need to be removed from the wound, alleviates pain and avoids the destruction of new tissues.

**FIGURE 4:**
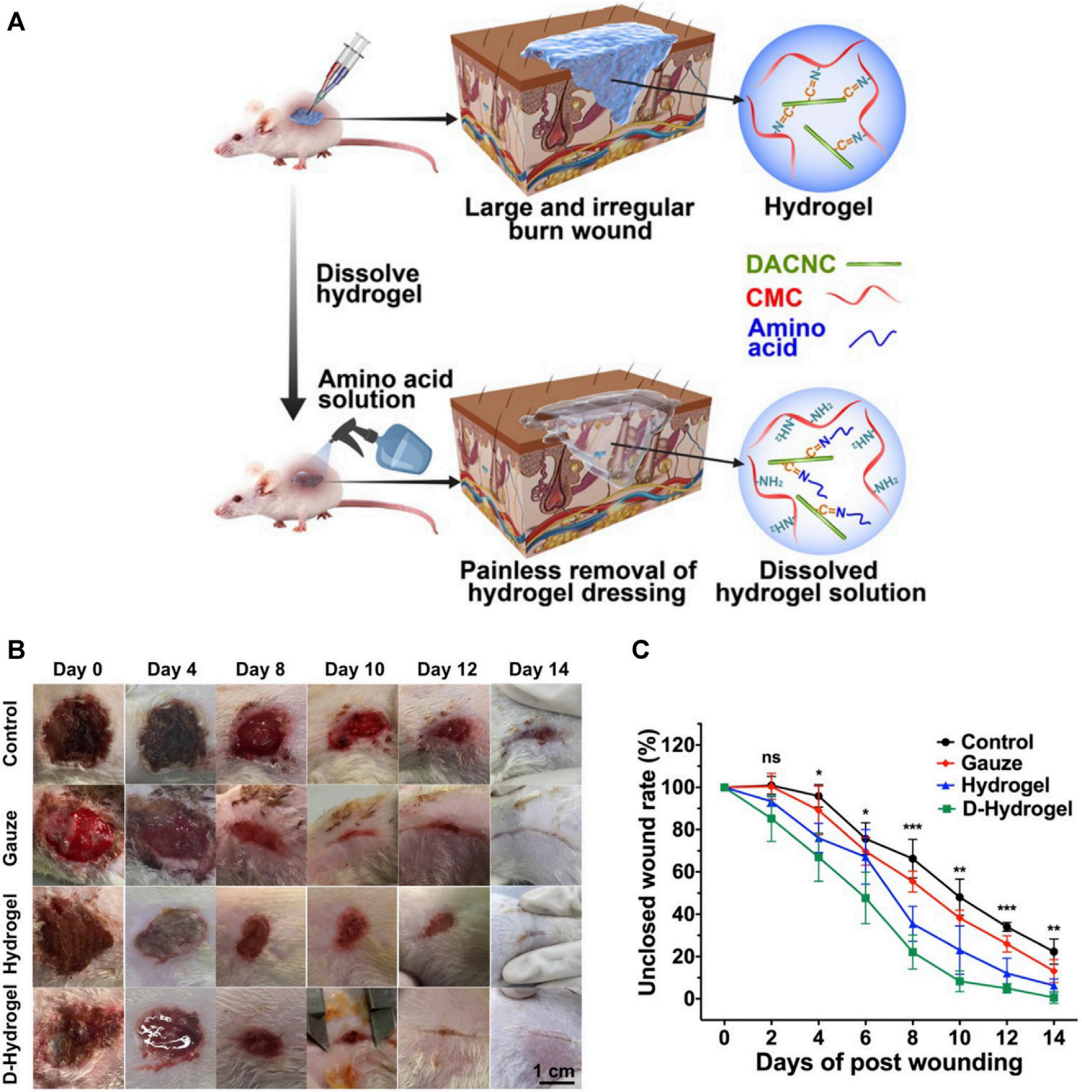
“On demand” dissolvable hydrogels for the healing of deep partial-thickness burns. **(A)** How on-demand dissolvable self-healing hydrogels are used to treat wounds (schematic). **(B)** Representative images of wound sites in each treatment group over time. **(C)** Unclosed wound area rate of initial wound as a function of time. ns *p* > 0.05, *p ≤ 0.05, **p ≥ 0.01, ***p ≤ 0.001. Reproduced with permission from (Huang et al., 2018).

Researchers have crosslinked lysine-based dendron and a polyethylene glycol (PEG)-based crosslinker to develop a dissolvable dendritic thioester hydrogel dressing for second-degree burns (Konieczynska et al., 2016). This hydrogel is unique because it can be removed readily through a thiol–thioester exchange reaction when needed. Other researchers have combined water-soluble carboxymethyl chitosan (a natural polymer) with rigid dialdehyde-modified cellulose nanocrystal to form self-healing nanocomposite hydrogels (Huang et al., 2018). When the hydrogel was injected into an irregular burn wound, it reformed instantly into an integrated piece, filling the wound area. When the dressing must be changed or removed, an amino-acid solution can be used to dissolve the hydrogel, allowing painless dressing removal (Figure 4). Wound bandages are very rigid, lack porosity, have low mechanical strength, poor affinity, and cannot resist bacterial invasion. Some researchers have prepared a composite bandage by combining nano-ZnO with keratin–chitosan hydrogels. These nano-ZnO-containing bandages have been shown to accelerate the construction of skin cells and collagen formation in Sprague–Dawley rats, thereby enhancing wound healing and overcoming the shortcomings of medical bandages (Huang et al., 2017).

### 4.2 Antibacterial Hydrogel Dressings

Wounds have been treated with local antibiotics since the 1960s (Heggers et al., 1991) and, subsequently, silver sulfadiazine(SSD) (Moyer et al., 1965; Fox, 1968). Early resection and transplantation have reduced the prevalence of infection and mortality of burn patients. However, the sepsis caused by infection with Gram-positive and Gram-negative bacteria is a prevalent trigger of death after a burn (Bang et al., 2002; Church et al., 2006). Given that bacterial resistance is widespread and it is difficult for systemic antibiotics to reach local wounds, control of local infection is the key to reducing the mortality from burns.

Often, burn-related deaths are closely related to burn infection. The latter occurs primarily through wound infection, and carries a mortality prevalence of 75–85%. Antibacterial hydrogel burn dressings can not only absorb wound fluid and maintain the moist environment of the wound, but also isolate the external pollution and prevent the wound from direct contact with the external environment. More importantly, antibacterial hydrogel burn dressings effectively reduce wound bacterial colonization and infection and speed up the healing process. Antimicrobial therapy of wounds is a critical component of burn treatment, and the development of antimicrobial hydrogel dressings is an ongoing research focus. A new type of hydrogel loaded with SSD and essential fibroblast growth factor and optimized based on SSD cream was found to not irritate skin or eyes (Chakrabarti et al., 2018). A thermo-sensitive methylcellulose hydrogel containing silver oxide nanoparticles (NPs) was also prepared as an injectable hydrogel and demonstrated an excellent antibacterial effect on the wound surface (Kim et al., 2018). Grolman and colleagues (Grolman et al., 2019) prepared agarose hydrogels containing high concentrations of minocycline or gentamicin. They demonstrated the stability of the two antibiotics in the hydrogels for ≥7 days in a porcine model of burns. The agarose minocycline hydrogel was as effective as the commonly used SSD cream in reducing burn depth and the number of bacteria. Demirci and coworkers (Demirci et al., 2015) prepared a novel antimicrobial carbopol hydrogel composed of boron and multiblock copolymers. This hydrogel not only increased wound healing through fibroblast activity but also induced angiogenesis and showed significant antimicrobial effects on bacteria, yeasts, and fungi. That study was the first to show that boron-containing hydrogels could treat burn wounds effectively.

**FIGURE 5:**
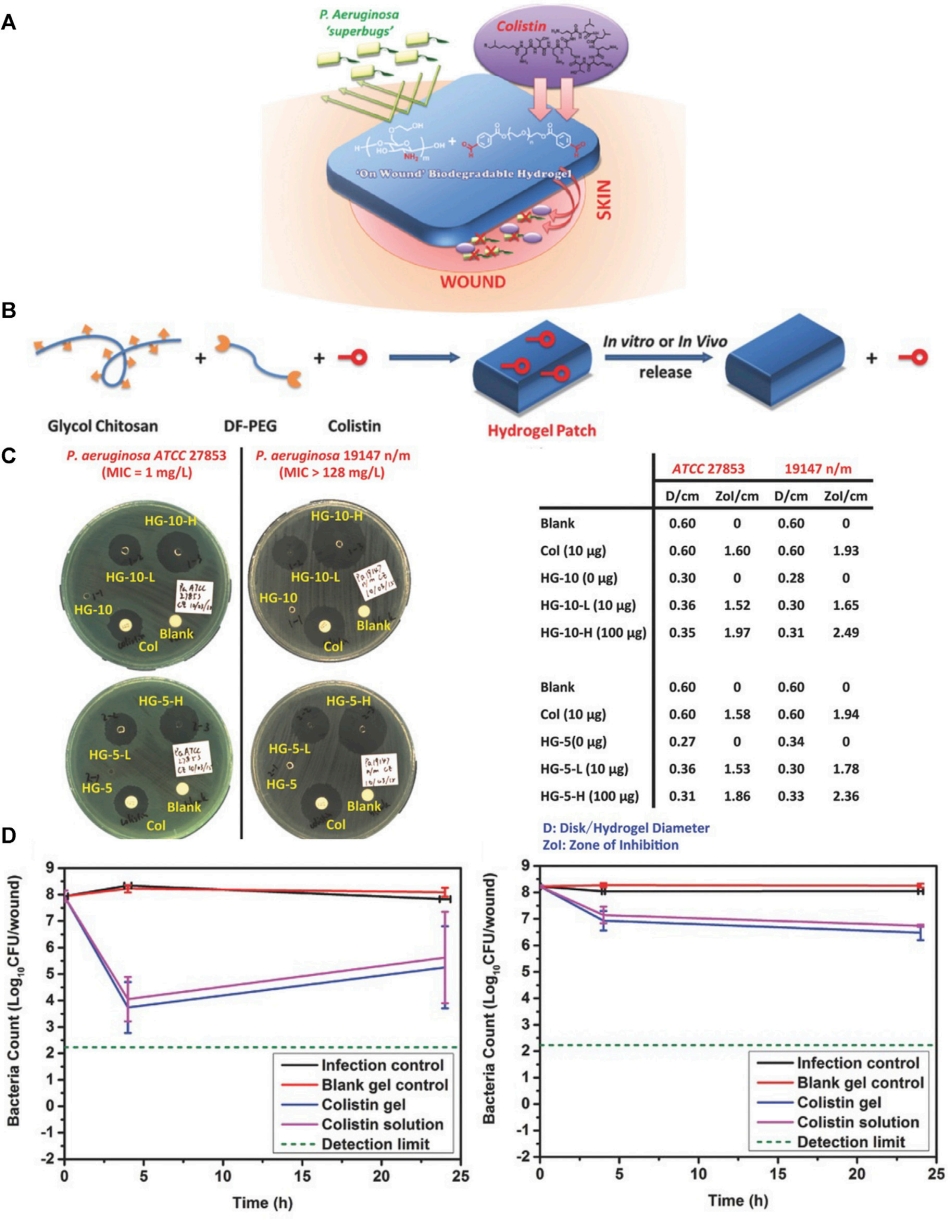
Hydrogel-based localized release of colistin for antimicrobial treatment of infections from burn wounds. **(A)** Treatment of a burn wound with a colistin-loaded hydrogel (schematic). **(B)** Synthesis of a colistin-containing hydrogel (schematic). **(C)** Disk diffusion assay of a colistin-loaded hydrogel against colistin-sensitive (left) and colistin-resistant (right) *P. aeruginosa* strains **(D)** Test of colistin-loaded hydrogel against colistin-sensitive (left) and colistin-resistant (right) strains of *P. aeruginosa* in a model of burn infection. Reproduced with permission from (Zhu et al., 2017).

Methicillin-resistant *Staphylococcus aureus* (MRSA) is a common colonizing bacterium in burn wounds worldwide, and is associated with high morbidity and mortality. Compared with systemic antibiotic treatment, antimicrobial hydrogel dressings avoid the effects on the whole body and maintain a higher drug concentration at the infection site. Chhibber and collaborators Chhibber et al. (2020) developed a chitosan-based hydrogel antibiofilm agent for local administration of moxifloxacin to wounds. Zhu et al. (2018) prepared heme-rich Dex-HA hydrogels that inhibited MRSA and *Escherichia coli*. Dex-HA promoted cell re-epithelialization and enhanced ECM remodeling in a full-thickness burn-infection model in rats, and reflected a highly effective scar-inhibition effect. Thanusha et al. (2018) prepared a hydrogel platform comprising biopolymer gelatin, glycosaminoglycans (HA and chondroitin sulfate), asiatic acid (a triterpenoid), and NPs (zinc oxide and copper oxide). The resulting hydrogel could resist *E. coli* and *S. aureus* in second-degree burn wounds in rats, and promote wound re-epithelialization, collagen-fiber arrangement, and angiogenesis. New treatments are needed urgently for *Pseudomonas aeruginosa* infections of burn wounds caused by multidrug-resistant Gram-negative “superbugs”. Zhu and coworkers (Zhu et al., 2017) chemically reacted the amine groups in glycol chitosan and aldehyde-PEG and combined them with colistin (potent lipopeptide) to form self-healing hydrogels (Figure 5). This process increased the storage modulus of the colistin hydrogel from 1.3 to 5.3 kPa, enabled sustained release of colistin from the hydrogel, and maintained wound activity, colistin susceptibility, and microbial resistance. *P. aeruginosa* has almost the same lethality as that of natural colistin.

Numerous attempts have been made to develop new antimicrobial materials and ways of delivering antibiotics to develop innovative antimicrobial hydrogels. Sanchez and colleagues Sanchez et al. (2018) developed a transparent carbomer hydrogel (CbCipLid) that combines ciprofloxacin and lidocaine at neutral pH in a manner higher than its solubility to treat second-degree burns. CbCipLid hydrogel also provides immediate anesthesia for wounds. Oryan and coworkers (Oryan et al., 2018) explored the effect of type-I collagen hydrogel scaffolds carrying *Saccharomyces cerevisiae* on the healing of burn wounds. Topical application of probiotics has been shown to antagonize *Klebsiella pneumonia*, *P. aeruginosa*, *S. aureus*, and *Bacillus subtilis*. More importantly, probiotics have a positive effect on the severe inflammatory response to burns through immunomodulation. Ouyang and collaborators (Ouyang et al., 2018) demonstrated that chitosan-conjugated hydrogels containing peptides extracted from mariculture of tilapia exhibited antibacterial activity and promoted the proliferation and migration of cells, skin regeneration, and accelerated burn healing.

### 4.3 Tissue Regeneration Dressings

#### 4.3.1 Stem Cells-Loaded Hydrogel Dressings

Tissue engineering has entered the era of “regenerative medicine” (Auxenfans et al., 2015). Tissue engineering and regenerative medicine can be employed to promote healing and provide templates for the reconstruction of burn wounds. Use of stem cells to replace or repair severely damaged tissues (Auxenfans et al., 2014) and a combination of stem-cell technology and biomaterial engineering are keys to developing new dressings to treat burns (Hermans, 2011). Keratinocytes are essential for wound closure. Cytokine activation leads to keratinocyte migration, which leads to the closure and recovery of a vascular network (Bisson et al., 2013). Thus, hydrogel dressings containing keratinocytes may be clinically viable treatment options for burns (Idrus et al., 2014). Mohamad et al. (2019) introduced human epidermal keratinocytes and human dermal fibroblasts into thymic-free mice. After loading with hydrogel, collagen deposition on the treated burn wound was more obvious, which suggested an expanded application scope of BC/AA hydrogel.

Application of adult stem cells represents a major advance in treating severe burns (Lewis, 2013). Bone-marrow stem cells added to hydrogels can promote healing of burn wounds because they can transform into various types of skin cell (Badiavas, 2004). Ke et al. (2015) extracted polysaccharides from cold-tolerant orchids and the mesenchymal stem cells (MSCs) of Sprague–Dawley rats and used them to prepare hydrogels. When used for treating alkali burns to eyes in rats, the resulting hydrogels were found to significantly improve corneal epithelial recovery and reduce inflammation, neovascularization, and opacity after corneal healing. Alapure and coworkers (Alapure et al., 2018) implanted MSCs into biodegradable composite hydrogels made of unsaturated arginine-based polyesteramide and chitosan derivatives. When applied to treat third-degree burn wounds in mice, the composite hydrogels promoted re-epithelialization, formation of granulation tissue, and vascularization of wounds. Thus, these hydrogels can be used to supplement commonly used skin grafts and overcome some of the shortcomings of transplantation. Zhou and collaborators (Zhou et al., 2019) prepared a thermosensitive hydrogel consisting of human umbilical-cord MSC-conditioned medium, chitosan, collagen, and glycerophosphate (beta-oleophosphate). This hydrogel shortened the healing time of third-degree burn wounds, promoted the regeneration of granulation tissue, and inhibited the proliferation of scar tissue.

**FIGURE 6:**
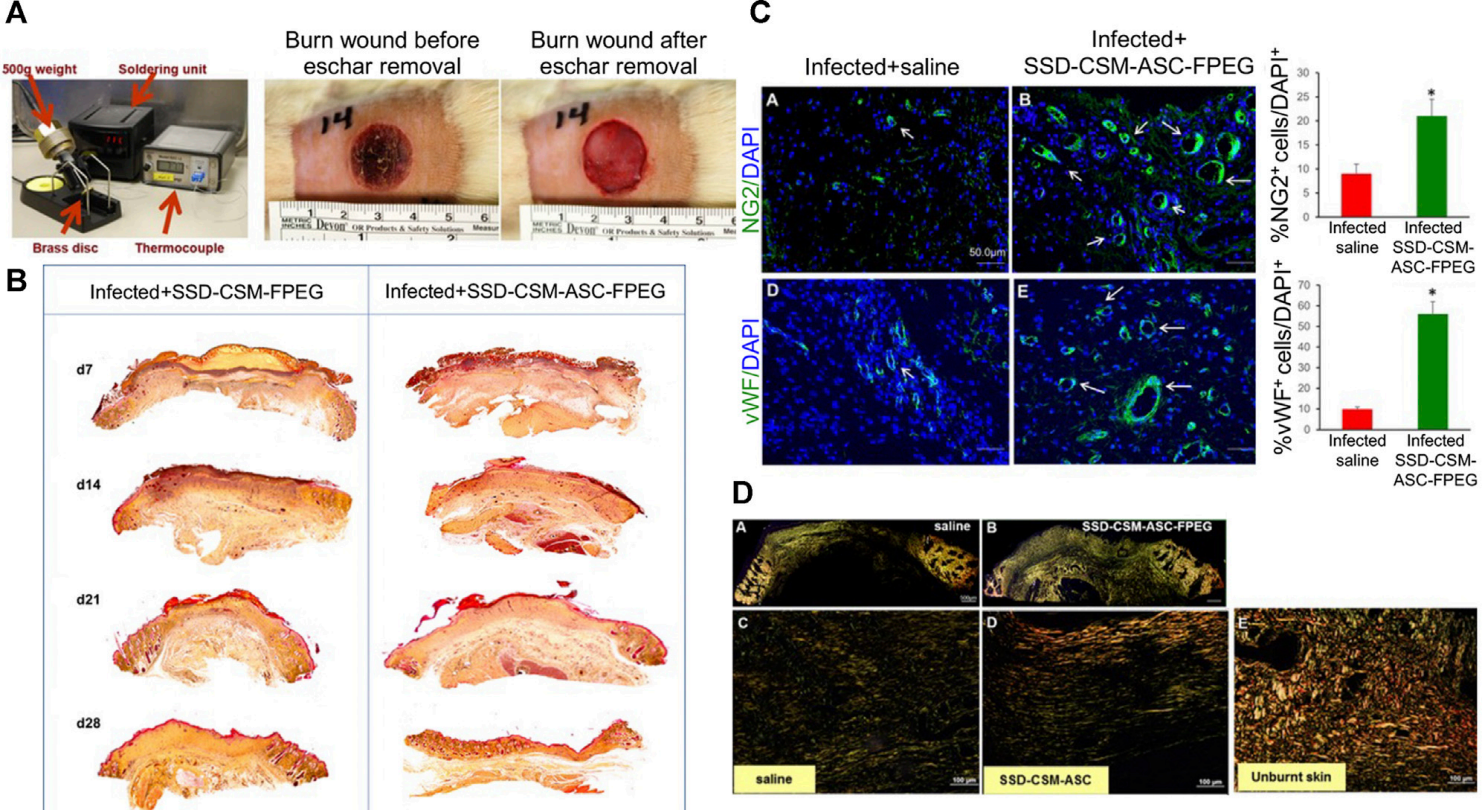
Delivery of silver sulfadiazine and adipose-derived stem cells using fibrin hydrogel improves infected burn wounds. **(A)** Photographs of the burn device and burn wound. **(B)** Burn wounds treated with SSD-CSM-ASC-FPEG had significantly thicker granulation tissue than those treated with SSD-CSM-FPEG. **(C)** SSD-CSM-ASC-FPEG facilitates neo-vascularization on day-21. *p* < 0.05. **(D)** Maturation of collagen. Picrosirius staining images on day-28. Reproduced with permission from (Banerjee et al., 2019).

Stem cells from hair follicles have also been incorporated into products that can generate a stratified epidermis on human burn wounds (Kim et al., 2007; Trottier et al., 2008). The possibility of making epidermal skin grafts with hair-follicle stem cells is under development. Adipose stem cells promote paracrine activation of host cells by secreting growth factors, producing epidermal, dermal, and subcutaneous layers, and accelerating re-epithelialization (Bey et al., 2010; Natesan et al., 2011). Usually, discarded human adipose stem cells can be separated from tissues and produce a three-layer vascularized structure (Chan et al., 2012). Banerjee et al. (2019) prepared a PEGylated fibrin hydrogel containing SSD chitosan microspheres (SSD-CSM-FPEG) and implanted adipose stem cells into this hydrogel (Figure 6). SSD-CSM-FPEG has been shown to reduce infections in a *P. aeruginosa* model of contact burns on the backs of rats as well as promote angiogenesis and improve matrix remodeling.

Treatment of burn wounds aims to achieve wound closure through skin regeneration rather than skin repair. Therefore, temporary wound dressings for skin healing are being replaced by temporary scaffolds or regenerative templates. The exciting results obtained from using non-embryonic stem cells have stimulated interest in the application and exploration of hydrogel dressings carrying stem cells.

#### 4.3.2 Wound Healing-Promoting Hydrogel Dressings

**FIGURE 7:**
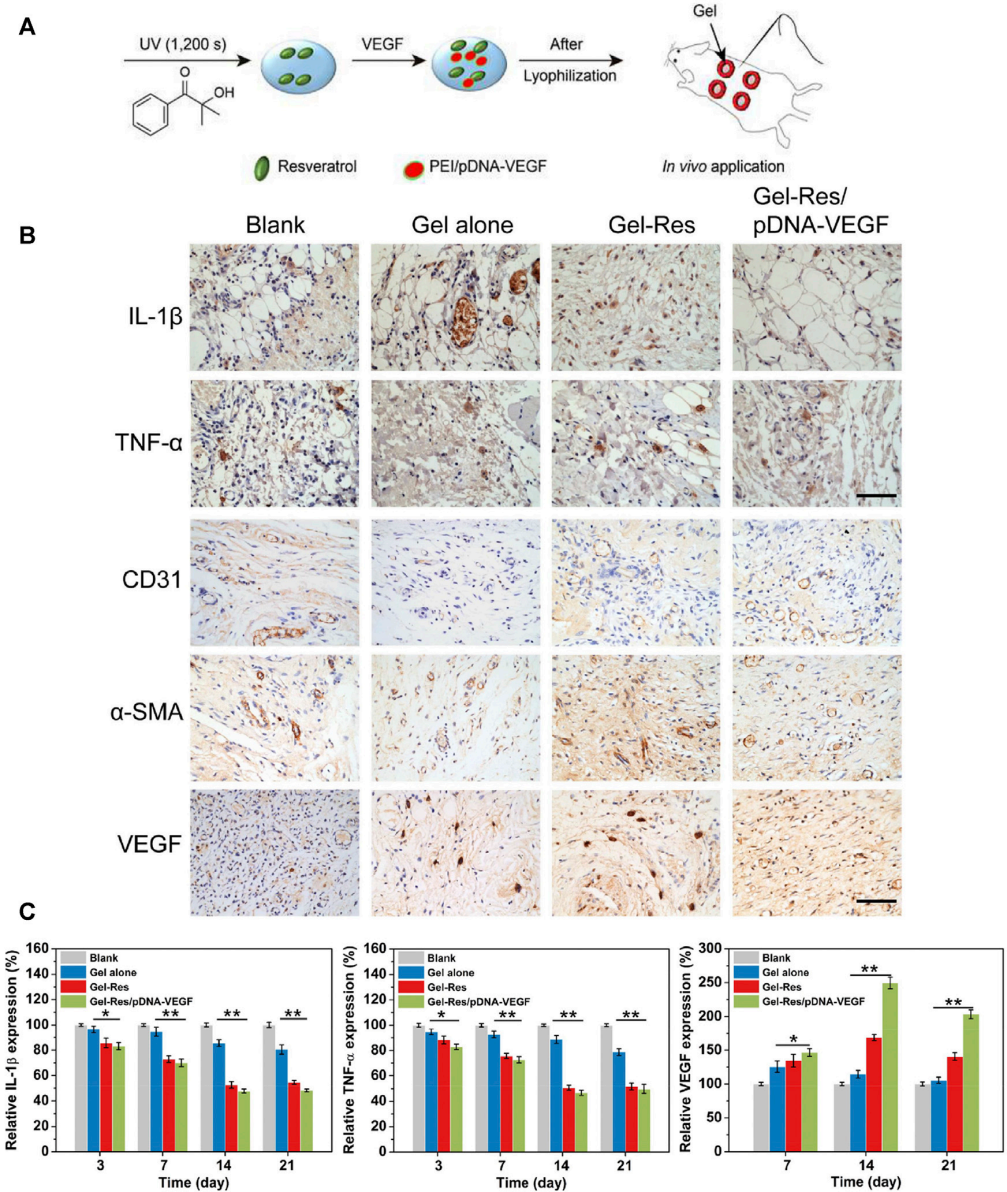
*In situ*-formed anti-inflammatory hydrogel loaded with plasmid DNA encoding VEGF for healing of burn wounds. **(A)** Application of this hydrogel in a model of a splinted excisional wound (schematic). **(B)** Immunohistochemical staining showing expression of the proinflammatory cytokines IL-1*β* and TNF-*α*. **(C)** Immunohistochemical staining showing expressions of the angiogenic factors VEGF, CD31, and *α*-SMA. Scale bar = 100 μm. **(D)** RT-qPCR of mRNA expression for inflammatory and angiogenic factors (IL-1β, TNF-α, VEGF). **p ≤ 0.01, *p ≤ 0.05. Reproduced with permission from (Wang et al., 2019).

New treatments are being developed thanks to deepening understanding of wound-healing mechanisms. The epidermal growth factor (EGF) receptor is a target during burn healing. Exogenous application of EGF can accelerate re-epithelialization. Understanding of the EGF-mediated pathway during burn healing represents a breakthrough in exogenous treatment. Wang et al. (2019) produced a composite hydrogel composed of modified HA, Dex, beta-cyclodextrin, resveratrol, and vascular EGF plasmids (Figure 7). The resulting hydrogel significantly inhibited the inflammatory response, promoted microvessel formation, and accelerated the healing of burn wounds. Yan and coworkers (Yan et al., 2012) explored the effect of recombinant human granulocyte/macrophage colony-stimulating factor (rhGM-CSF) hydrogel on wound healing in 93 patients with a deep partial-thickness burn. The rhGM-CSF hydrogel was better than the control group in terms of wound-healing rate, healing time, wound exudation, pus score, and secretion score. Those findings suggested that rhGM-CSF hydrogel could support burn healing effectively. Thus, molecules or genes can be manipulated in hydrogel dressings to enhance the desired effects (i.e., introduce “positive” cytokines and suppress “negative” factors) (Atiyeh et al., 2005). Development of hydrogel dressings containing pro-wound-healing characteristics for burns is an active area of research.

### 4.4 Other Types of Hydrogel Dressings

The human amniotic membrane avoids large-area dressings, reduces the pain associated with dressing changes, and accelerates epithelial-cell regeneration when treating burn wounds, especially if religious sensitivities hinder application of bovine, porcine, or cadaveric skin (Gajiwala and Lobo Gajiwala, 2003). Rana and colleagues Rana et al. (2020) prepared an acellular hydrogel composed of human amniotic membrane and rabbit collagen that significantly promoted wound healing and re-epithelialization in a model of second-degree burns. Amniotic membranes and collagen-based hybrid hydrogels are inexpensive and easy to manufacture.

Honey is a natural material with excellent biocompatibility. It has shown promising results in treatment of burn wounds. Zohdi and collaborators (Mohd Zohdi et al., 2012) fabricated a crosslinked hydrogel containing Malaysian honey and demonstrated that it had a significant inhibitory effect on wound inflammation. Some scholars have found that hydrogels prepared with 75% honey had high activity against wound infections caused by *P. aeruginosa*, *S. aureus*, or *Klebsiella pneumoniae* (Mohd Zohdi et al., 2012). Some researchers have introduced sea cucumber (Zohdi et al., 2011) into hydrogels to develop a new crosslinked Gamat hydrogel dressing. The latter was shown to regulate the inflammatory response, stimulate the activation and proliferation of fibroblasts, significantly promote wound contraction and the rapid generation of a collagen-fiber network, and shorten the healing time effectively.

Nuutila and coworkers (Nuutila et al., 2020) combined a platform wound device (PWD) with a sodium-alginate hydrogel containing a high concentration of antibiotics. When wounds infected by *S. aureus*, *P. aeruginosa*, or *Acinetobacter baumannii* were covered with the hydrogel/PWD device, the covered wound had less bacteria along with less necrotic depth. The hydrogel/PWD could be used safely at high concentrations and applied effectively to burn infections of any size. In future management of burn wounds, auxiliary treatment devices will be developed to help monitor wound conditions, quantify micro-administration in real time, extract tissue fluid for biochemical examination, improve the labor cost of burn-wound care, and prevent nosocomial wound infection.

## 5 Conclusion and Outlook

Targeting many problems faced in the repair process of burn wounds, the functionalities of dressings can be specifically designed during the construction of hydrogel dressings. Burn injuries present many necrotic tissues, so guaranteeing wound debridement is a crucial prerequisite to promote burn wound healing. The hydrogel dressing itself has a particular debridement ability. However, autolytic debridement hydrogel and enzyme debridement hydrogel improved the debridement effect in burn treatment. Autolytic debridement hydrogels achieve the purpose of debridement by removing devitalized or necrotic tissues through many ways, such as softening, hydrolysis, and autolysis. But the disadvantage is the long debridement period. Selective enzyme debridement hydrogels can retain undamaged dermal tissue on the wound bed, accelerate the wound’s healing rate, and reduce scar formation, which has the disadvantage of higher fabrication cost.

Wound infection is another critical factor affecting wound healing, and the proliferation of bacteria and the secretion of enzymes can interfere with re-epithelialization and collagen synthesis. Therefore, hydrogel dressings must possess a particular antibacterial ability. Hydrogel dressings can achieve physical barrier sequestration of microorganisms after coverage of the wound, but sometimes this barrier is insufficient to control the extent of wound infection. The antibacterial properties of hydrogels can be further improved with the addition of antibiotics, antimicrobial agents, and the use of materials with antibacterial activity, reducing the risk of wound infection. Currently, the addition of broad-spectrum antibiotics or silver ions with antibacterial ability into hydrogel dressings is mostly adopted. But the application of broad-spectrum antibiotics increases the possibility of developing drug-resistant bacteria. In addition, the potential toxicity of silver ions limits its applications to some extent. Therefore, developing materials with antibacterial activity will be an important area of future research on hydrogel dressings.

Based on adequate debridement and limiting local inflammation, wound healing, and tissue regeneration, especially angiogenesis at the injury site. The introduction of growth factors and functional peptides with specific functions improves the tissue repair ability of hydrogel dressings and plays an essential role in promoting cell proliferation, differentiation, and angiogenesis, but has disadvantages such as higher cost and difficult to mass-produce.

During the nursing process of the burn wound, considering that burn injuries dermis stimulates nerve endings, the pain reaction brought during dressing change is much greater than other wounds. During the clinical dressing change, the replacement of conventional dressings often caused damage to the newly formed skin and tissues, increasing the suffering of patients. While the development of on-demand dissolving hydrogel brings a milder method of dressing replacement and avoids the secondary damage of newly formed tissues during the process of dressing change, which can relieve patient’s pain to a more considerable extent.

The limitations of traditional dressings, technological advances, and understanding of burn-wound healing have led to a massive expansion of the range of available burn dressings and promoted the development of new burn dressings (Zhang et al., 2020). Future research on hydrogel dressings for burns is expected to focus on inflammation regulation, infection control, stem cells, transplantation, biomarkers, factors affecting wound healing, and individualized treatment. The way to achieve this goal is to develop hydrogel dressings of multifunctional composite materials. Such materials will improve wound management (e.g., through infection control and dressing elasticity) and wound healing (e.g., epithelialization, collagen synthesis, vascularization, contraction) systematically, deliver active molecules to the sites of interest, and monitor healing to meet the different needs of all the processes involved in wound healing.

The future development trend of hydrogel dressing should be considered according to the clinical need for burn wound treatment. In the early stages of burn wound treatment, emphasis should be placed on considering the anti-infection properties of the hydrogel dressing, mainly to limit bacterial proliferation and control inflammatory factor levels. In the stage of wound repair, hydrogel dressings are required to provide a suitable microenvironment for granulation tissue, which should have the ability to promote skin tissue repair and consider the possibility of inhibiting scar formation.

Furthermore, combining hydrogel dressings with skin tissue engineering concepts can lead to better optimization of hydrogel dressings from a tissue repair perspective. Hydrogel dressings provide a network structure for tissue regeneration and act as a delivery system for cells and biochemical factors. During the repair process, better deposition of ECM around the scaffolds can be achieved through the gradual degradation of the hydrogel, ultimately achieving good integration of the newly formed tissue with the surrounding tissue, preventing scar formation and skin hypofunction.

Most studies on hydrogel dressings for wounds have focused on accelerating the rate of wound healing. However, rapid wound closure would lead to disordered deposition of collagen into the injury site, which is not beneficial for alleviating scar formation or restoring the inherent glands, hair follicles, nerve tissue of skin tissue. Therefore, in the treatment of burn wounds, it is essential to construct different functional hydrogel dressings according to the different stages of wound healing and ensure that the appropriate therapy is administered when appropriate.
